# Supplementary material for: Prognostic Implications of Blood Immune-Cell Composition in Metastatic Castration-Resistant Prostate Cancer
Source: Cancers (Basel). 2024 Jul 14;16(14):2535. doi: 10.3390/cancers16142535 (PMC11274568; doi:10.3390/cancers16142535)
Supplement: Supplementary file 1 [file cancers-16-02535-s001.zip › Supplementary Figure 1.pdf]

Supplementary Figure 1. Kaplan−Meier Estimator for different types of immune cells.

This figure presents the Kaplan−Meier survival curves for various types of immune cells, providing a visual representation of their impact on overall survival. The analysis includes different immune cell types such as monocytes, CD8 T-cell lymphocytes, and others, highlighting their prognostic significance. Each subplot within the figure demonstrates the survival probabilities over time for patients stratified by high and low levels of these immune cells. The data reveal important insights into how specific immune cell populations correlate with patient outcomes, emphasizing the role of immune cell composition in prognostication

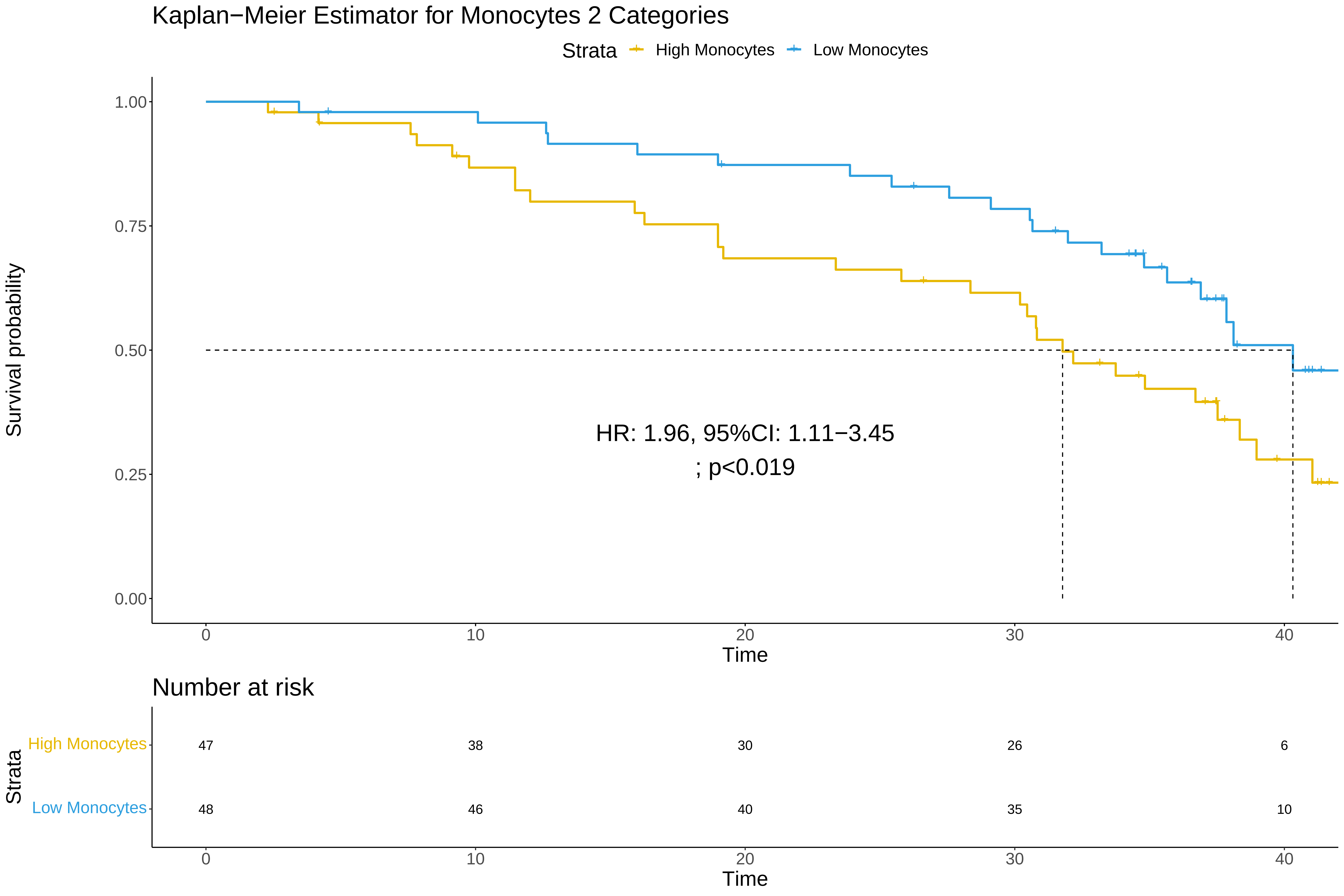
Kaplan−Meier Estimator for Monocytes 2 Categories
Strata
High Monocytes
Low Monocytes
Survival probability
1.00
0.75
0.50
0.25
0.00
HR: 1.96, 95%CI: 1.11−3.45
; p<0.019
0
10
20
30
40
Time
Number at risk
Strata
High Monocytes 47 38 30 26 6
Low Monocytes 48 46 40 35 10
0
10
20
30
40
Time

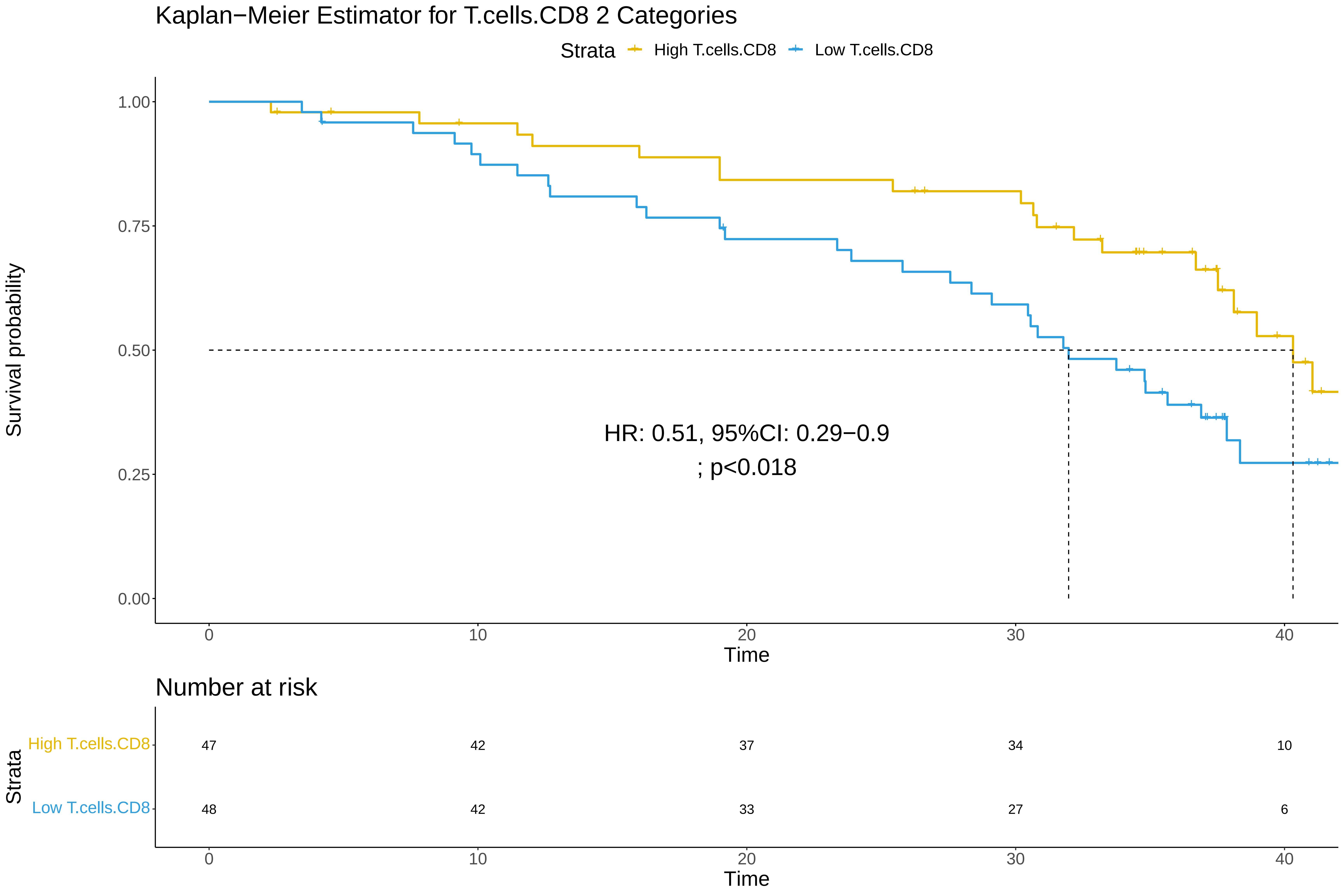
Kaplan−Meier Estimator for T.cells.CD8 2 Categories
Strata
High T.cells.CD8
Low T.cells.CD8
Survival probability
1.00
0.75
0.50
0.25
0.00
HR: 0.51, 95%CI: 0.29−0.9
; p<0.018
0
10
20
30
40
Time
Number at risk
Strata
High T.cells.CD8 47 42 37 34 10
Low T.cells.CD8 48 42 33 27 6
Time

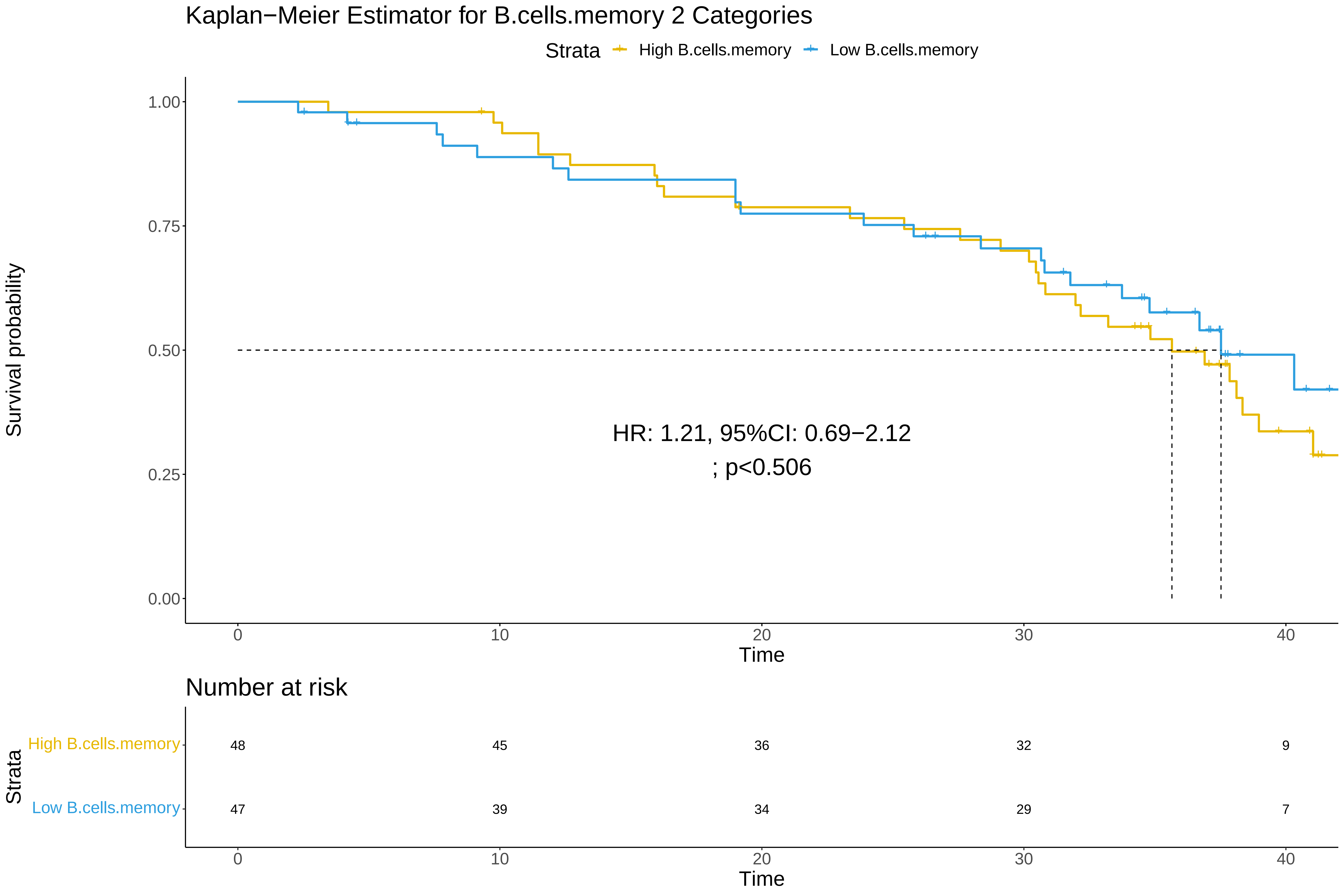

Kaplan−Meier Estimator for B.cells.memory 2 Categories
Strata
High B.cells.memory
Low B.cells.memory
Survival probability
1.00
0.75
0.50
0.25
0.00
HR: 1.21, 95%CI: 0.69−2.12
; p<0.506
0
10
20
30
40
Time
Number at risk
Strata
High B.cells.memory
48
45
36
32
9
Low B.cells.memory
47
39
34
29
7
0
10
20
30
40
Time

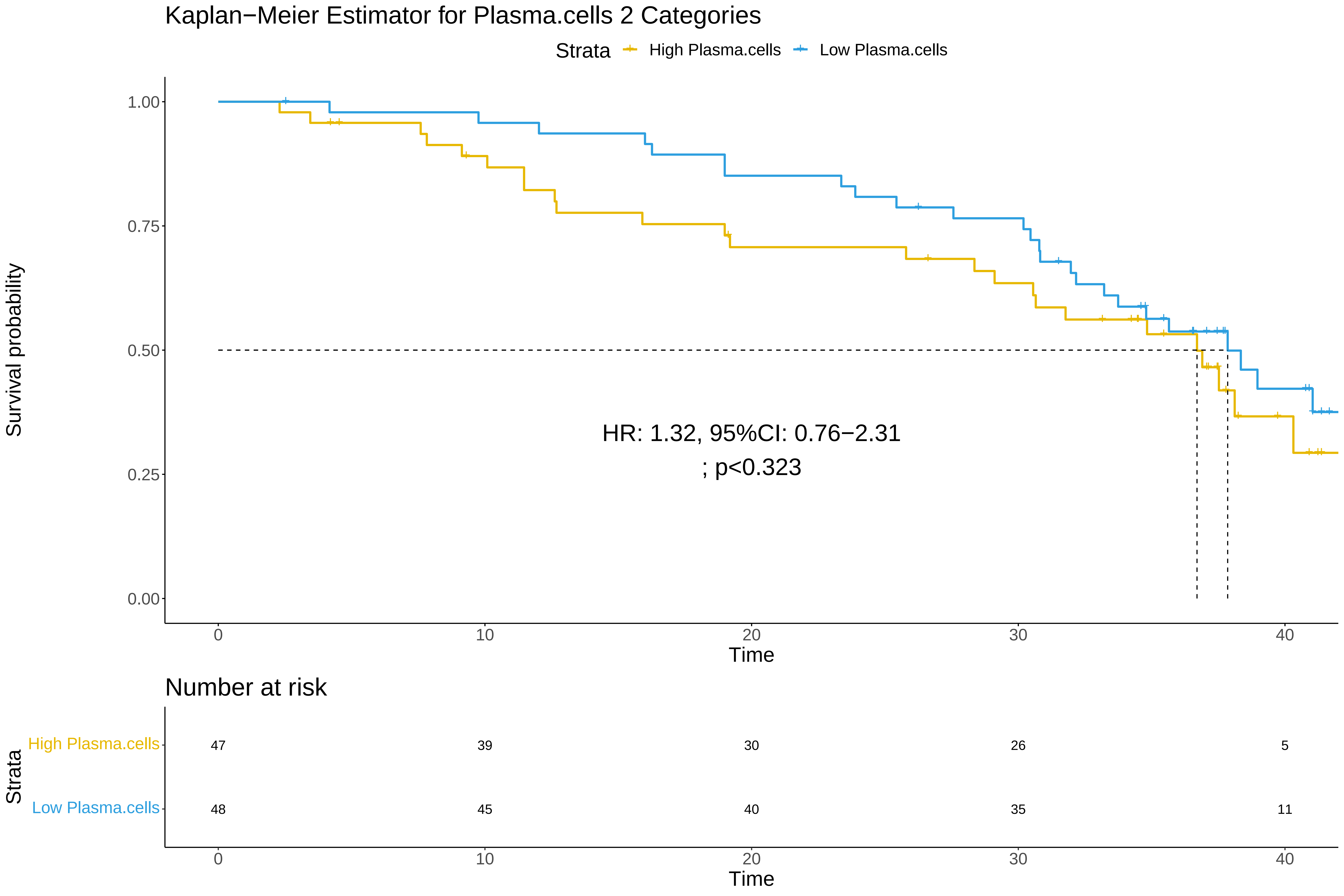

Kaplan−Meier Estimator for Plasma.cells 2 Categories
Strata
High Plasma.cells
Low Plasma.cells
Survival probability
1.00
0.75
0.50
0.25
0.00
HR: 1.32, 95%CI: 0.76−2.31
; p<0.323
0
10
20
30
40
Time
Number at risk
Strata
High Plasma.cells
47
39
30
26
5
Low Plasma.cells
48
45
40
35
11
0
10
20
30
40
Time

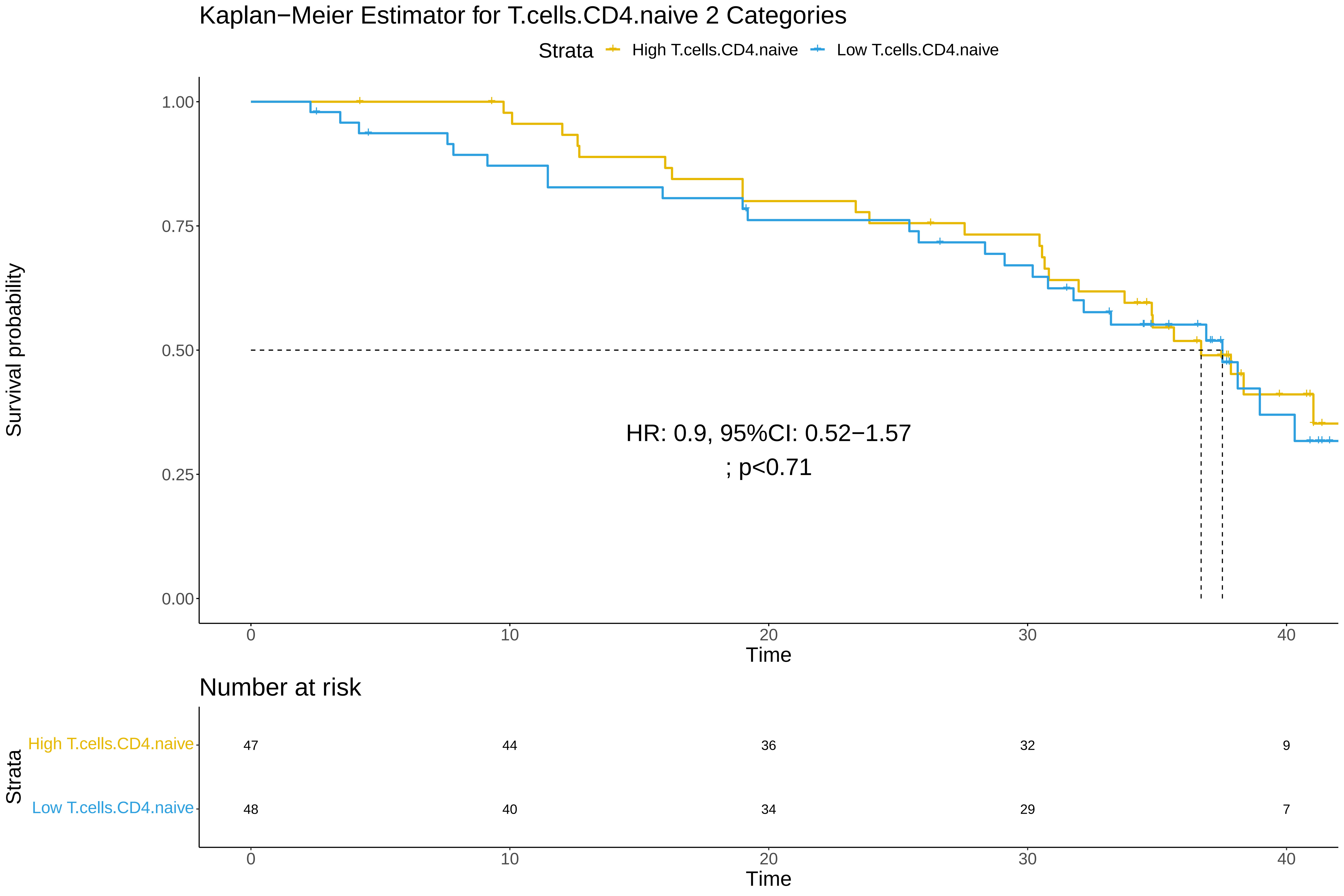

Kaplan−Meier Estimator for T.cells.CD4.naive 2 Categories
Strata
High T.cells.CD4.naive
Low T.cells.CD4.naive
Survival probability
1.00
0.75
0.50
0.25
0.00
HR: 0.9, 95%CI: 0.52−1.57
; p<0.71
0
10
20
30
40
Time
Number at risk
Strata
High T.cells.CD4.naive
Low T.cells.CD4.naive
47
44
36
32
9
48
40
34
29
7
0
10
20
30
40
Time

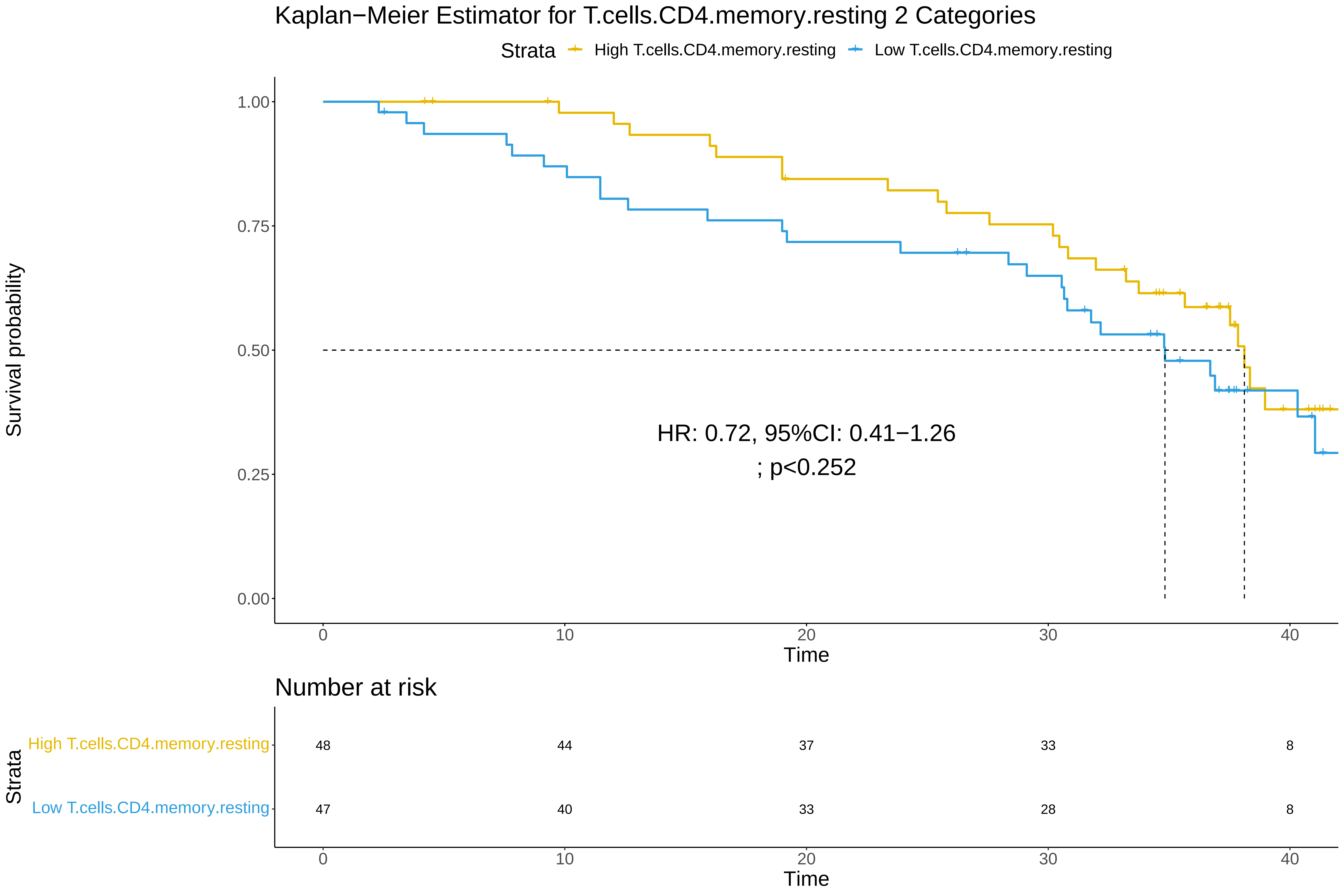
Kaplan−Meier Estimator for T.cells.CD4.memory.resting 2 Categories
Strata
High T.cells.CD4.memory.resting
Low T.cells.CD4.memory.resting
Survival probability
1.00
0.75
0.50
0.25
0.00
HR: 0.72, 95%CI: 0.41−1.26
; p<0.252
0
10
20
30
40
Time
Number at risk
Strata
High T.cells.CD4.memory.resting
48
44
37
33
8
Low T.cells.CD4.memory.resting
47
40
33
28
8
0
10
20
30
40
Time

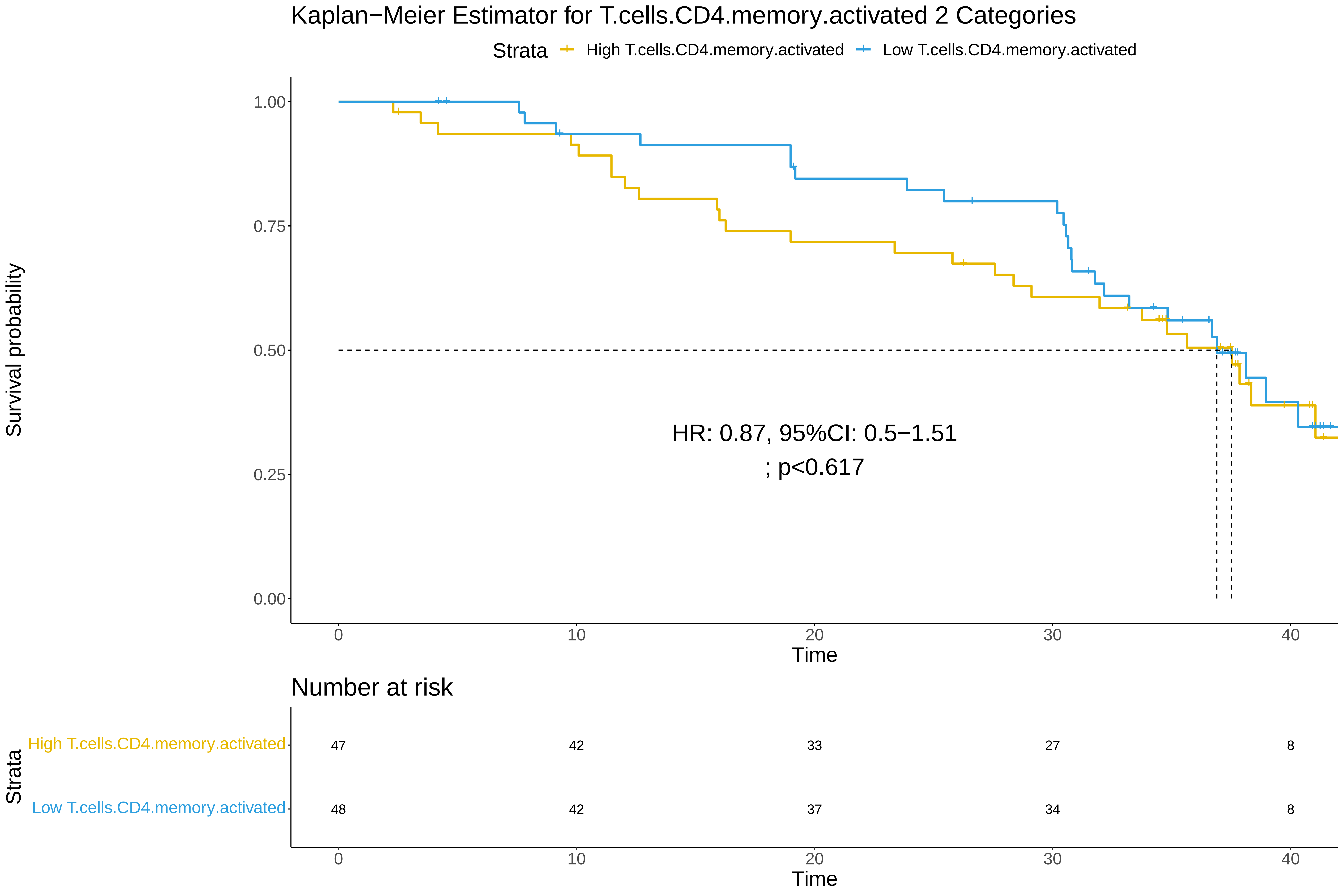

Kaplan−Meier Estimator for T.cells.CD4.memory.activated 2 Categories
Strata
High T.cells.CD4.memory.activated
Low T.cells.CD4.memory.activated
Survival probability
1.00
0.75
0.50
0.25
0.00
HR: 0.87, 95%CI: 0.5−1.51
; p<0.617
0
10
20
30
40
Time
Number at risk
Strata
High T.cells.CD4.memory.activated
47
42
33
27
8
Low T.cells.CD4.memory.activated
48
42
37
34
8
0
10
20
30
40
Time

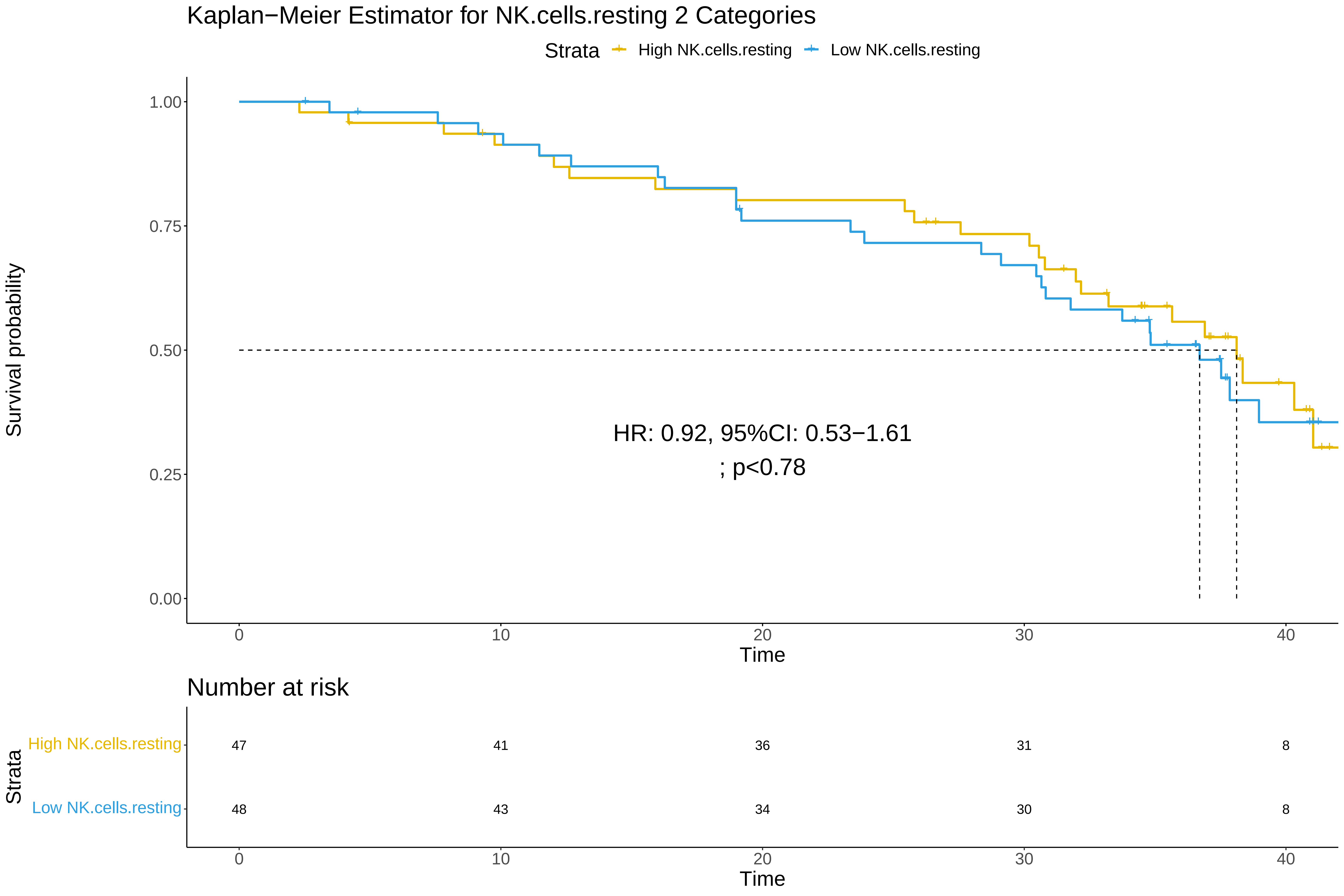

Kaplan−Meier Estimator for NK.cells.resting 2 Categories
Strata
High NK.cells.resting
Low NK.cells.resting
Survival probability
1.00
0.75
0.50
0.25
0.00
HR: 0.92, 95%CI: 0.53−1.61
; p<0.78
0
10
20
30
40
Time
Number at risk
Strata
High NK.cells.resting 47 41 36 31 8
Low NK.cells.resting 48 43 34 30 8
0
10
20
30
40
Time

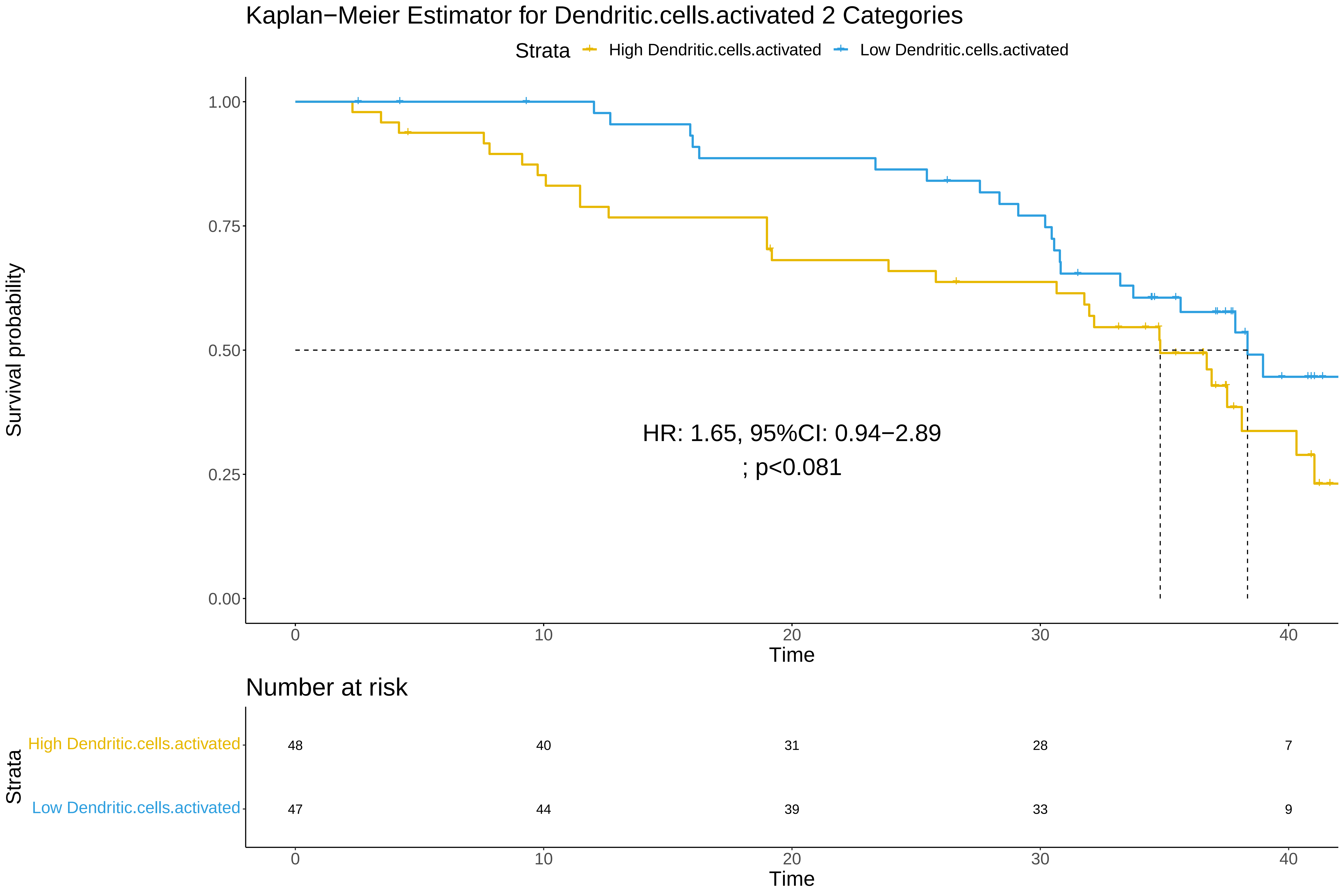

Kaplan−Meier Estimator for Dendritic.cells.activated 2 Categories
Strata
High Dendritic.cells.activated
Low Dendritic.cells.activated
Survival probability
1.00
0.75
0.50
0.25
0.00
HR: 1.65, 95%CI: 0.94−2.89
; p<0.081
0
10
20
30
40
Time
Number at risk
Strata
High Dendritic.cells.activated
48
40
31
28
7
Low Dendritic.cells.activated
47
44
39
33
9
0
10
20
30
40
Time

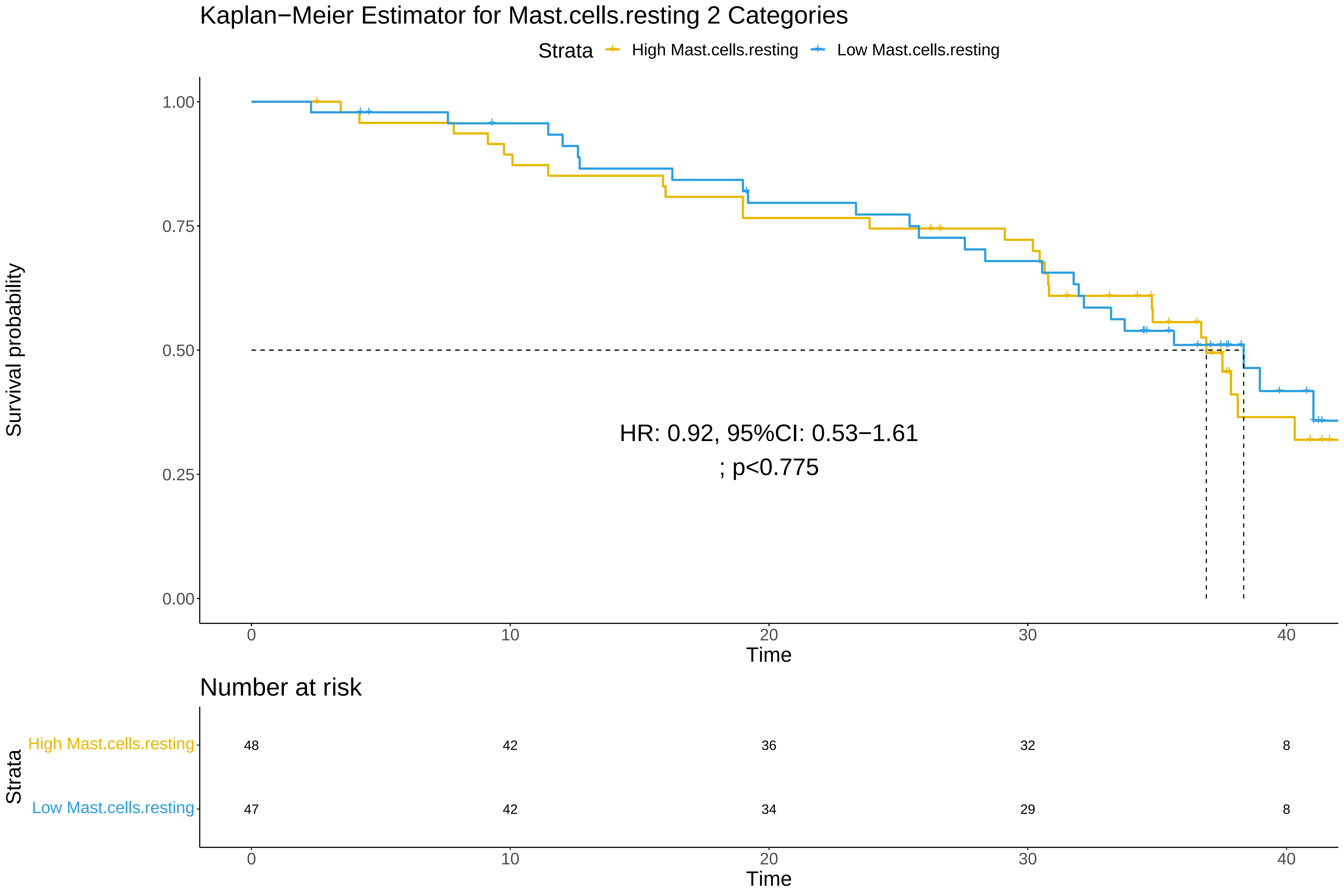

Kaplan−Meier Estimator for Mast.cells.resting 2 Categories
Strata
High Mast.cells.resting
Low Mast.cells.resting
Survival probability
1.00
0.75
0.50
0.25
0.00
HR: 0.92, 95%CI: 0.53−1.61
; p<0.775
0
10
20
30
40
Time
Number at risk
Strata
High Mast.cells.resting
48
42
36
32
8
Low Mast.cells.resting
47
42
34
29
8
0
10
20
30
40
Time

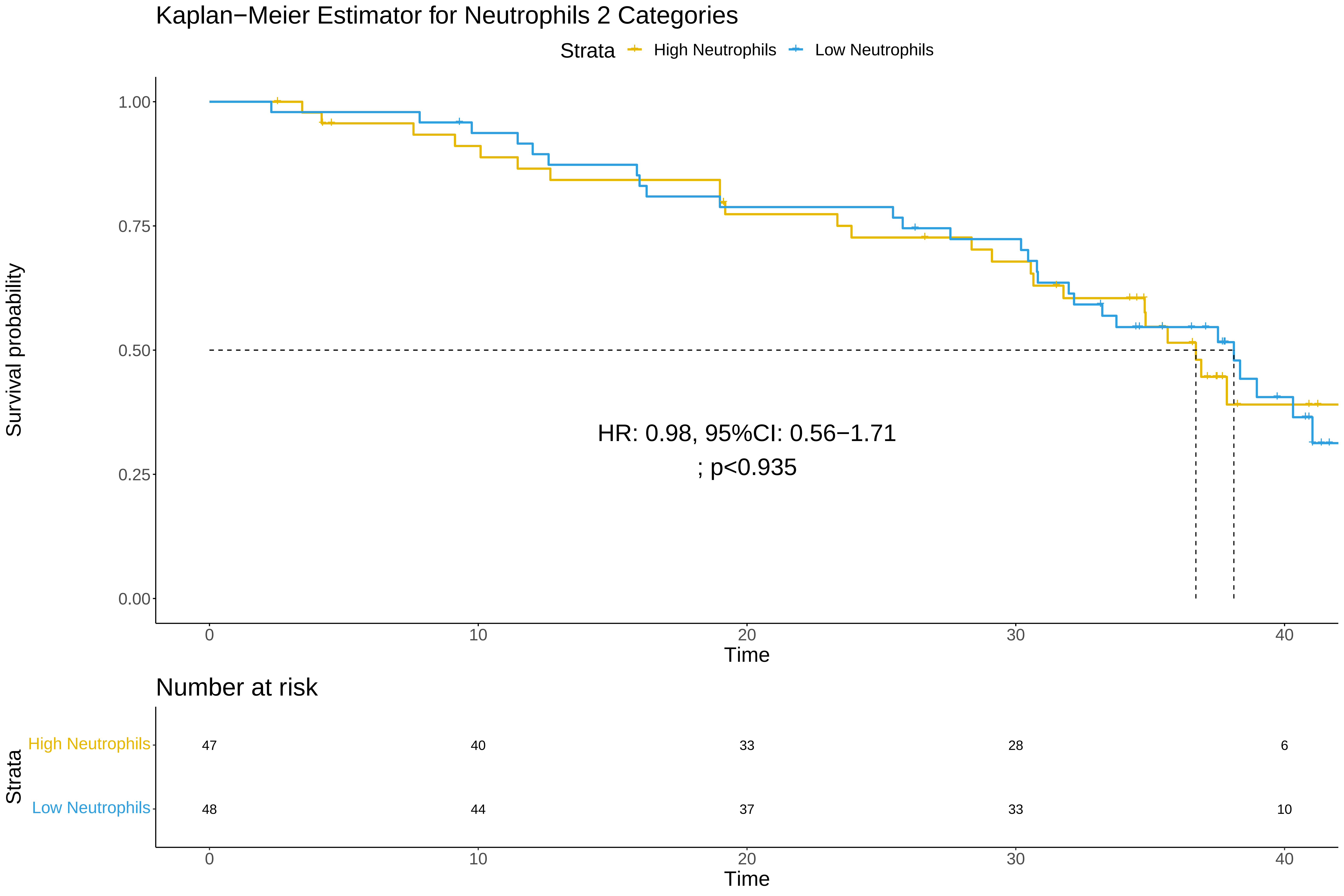

Kaplan−Meier Estimator for Neutrophils 2 Categories
Strata
High Neutrophils
Low Neutrophils
Survival probability
1.00
0.75
0.50
0.25
0.00
0
10
20
30
40
Time
HR: 0.98, 95%CI: 0.56−1.71
; p<0.935
Number at risk
Strata
High Neutrophils
47
40
33
28
6
Low Neutrophils
48
44
37
33
10
0
10
20
30
40
Time
